# Geyser Sign: An Eruption of Fluid through the Acromioclavicular Joint in Chronic Rotator Cuff Degeneration

**DOI:** 10.5334/jbsr.3205

**Published:** 2023-07-03

**Authors:** Matthias Lembrechts

**Affiliations:** 1 UZ Leuven, BE

**Keywords:** Rotator cuff degeneration, acromioclavicular joint degeneration, MR arthrogram

## Abstract

**Teaching Point:** Leakage of fluid or intra-articular contrast from the subacromial bursa through the acromioclavicular joint, also known as the ‘geyser sign’, is an uncommon presentation of chronic rotator cuff injury and acromioclavicular joint degeneration.

## Case History

**Figure 1:**
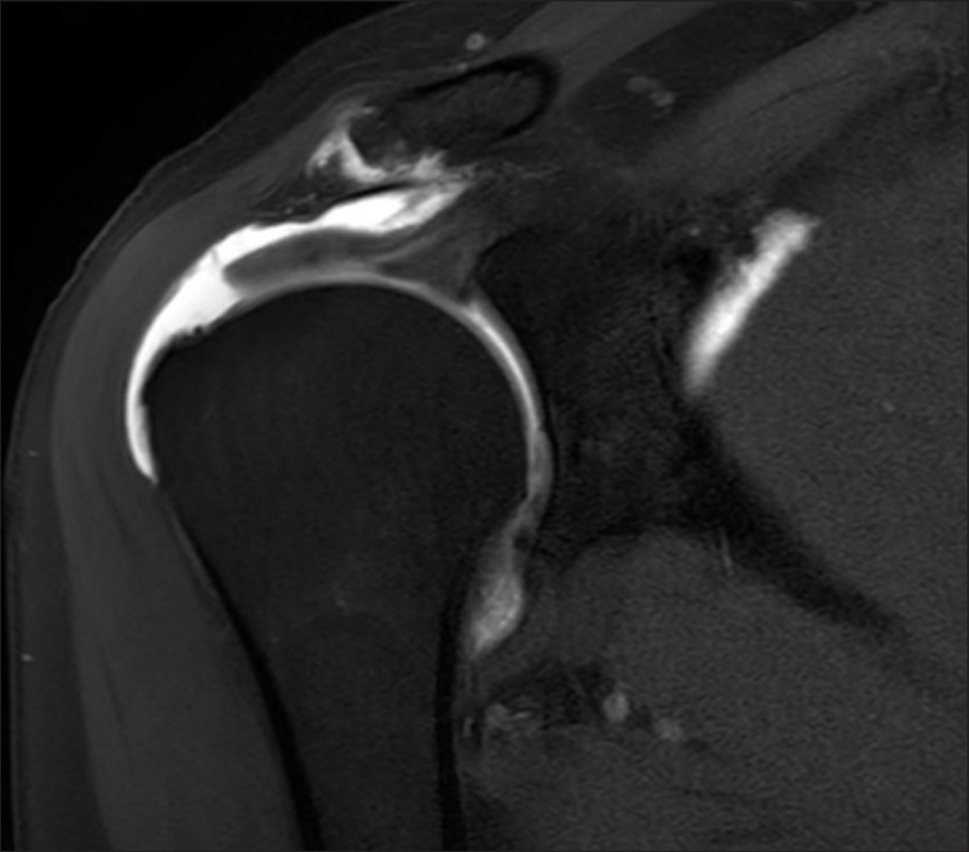


**Figure 2:**
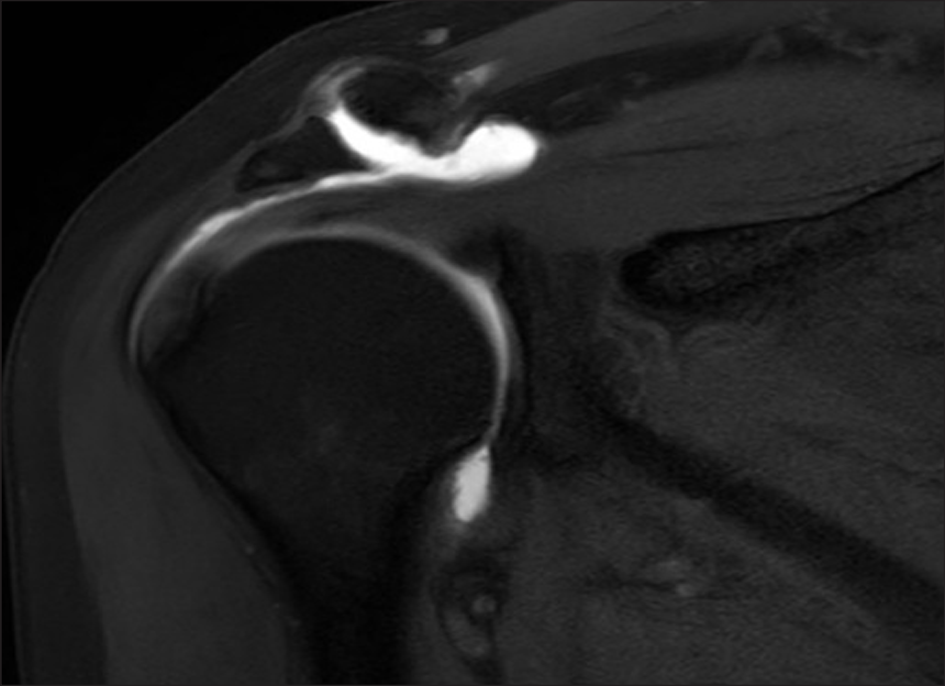


**Figure 3:**
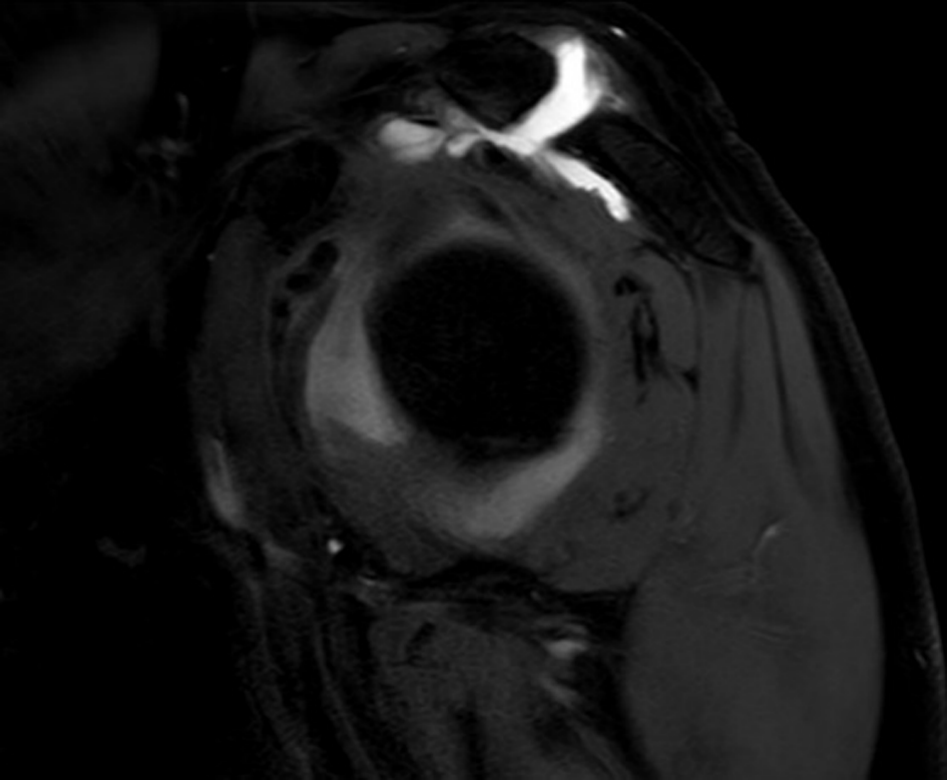


A 62-year-old man known with chronic rotator cuff pathology underwent a magnetic resonance (MR) arthrogram to assess the condition of the rotator cuff. The examination showed a full-thickness tear in the anterior segment of the supraspinatus tendon with leakage of intra-articular contrast to the subacromial bursa as well as chronic degeneration of the acromioclavicular joint (Figure 1). The contrast fluid extends through the acromioclavicular joint to form a small supraclavicular collection, known as ‘geyser sign’ (Figures 2, 3).

## Comments

Rotator cuff injury and acromioclavicular joint degeneration are common pathologies encountered in daily practice. The prevalence increases with age. The geyser sign is an uncommon presentation of chronic rotator cuff and acromioclavicular. It was first described in 1984 by Craig and can be observed on ultrasound (as a type 2 acromioclavicular cyst), arthrography and magnetic resonance imaging (MRI). Due to chronic degeneration of the rotator cuff and the acromioclavicular joint, the inferior capsule of the acromioclavicular joint can disrupt and allow extension of fluid from the bursa subacromialis-subdeltoidea to ‘erupt’ above the acromioclavicular joint [1]. In a few cases a palpable mass is described, also known as a type 2 acromioclavicular joint cyst. Aspiration of the cyst or excision results in a high risk of recurrence. The treatment is aimed at rotator cuff repair with resection of the distal clavicle or in severe case shoulder arthroplasty.
